# Get Out of Jail: Managing an Entrapped Balloon Using Subintimal Plaque Modification

**DOI:** 10.1016/j.jaccas.2022.07.004

**Published:** 2022-10-05

**Authors:** Mohamed Nosair, Ahmad Hayek, Robert Avram

**Affiliations:** Montreal Heart Institute, University of Montreal, Montreal, Quebec, Canada

**Keywords:** complications, entrapment, external subintimal crushing, guidewire, percutaneous coronary intervention, subintimal plaque modification, CAG, coronary angiography, CTO, chronic total occlusion, PCI, percutaneous coronary intervention, RCA, right coronary artery, TIMI, Thrombolysis In Myocardial Infarction

## Abstract

Balloon entrapment is a potentially fatal complication of percutaneous coronary intervention. This report describes the use of subintimal plaque modification for the management of entrapped balloons. This technique, commonly done during chronic total occlusion angioplasty, was used successfully to retrieve the balloon. (**Level of Difficulty: Advanced.**)

## Central Illustration

**Figure:**
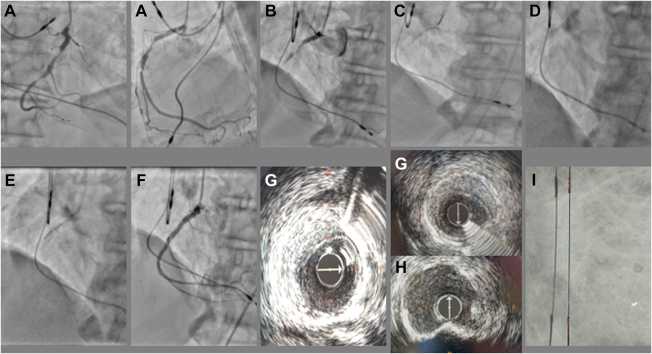

## History of Presentation

**Figure 1:**
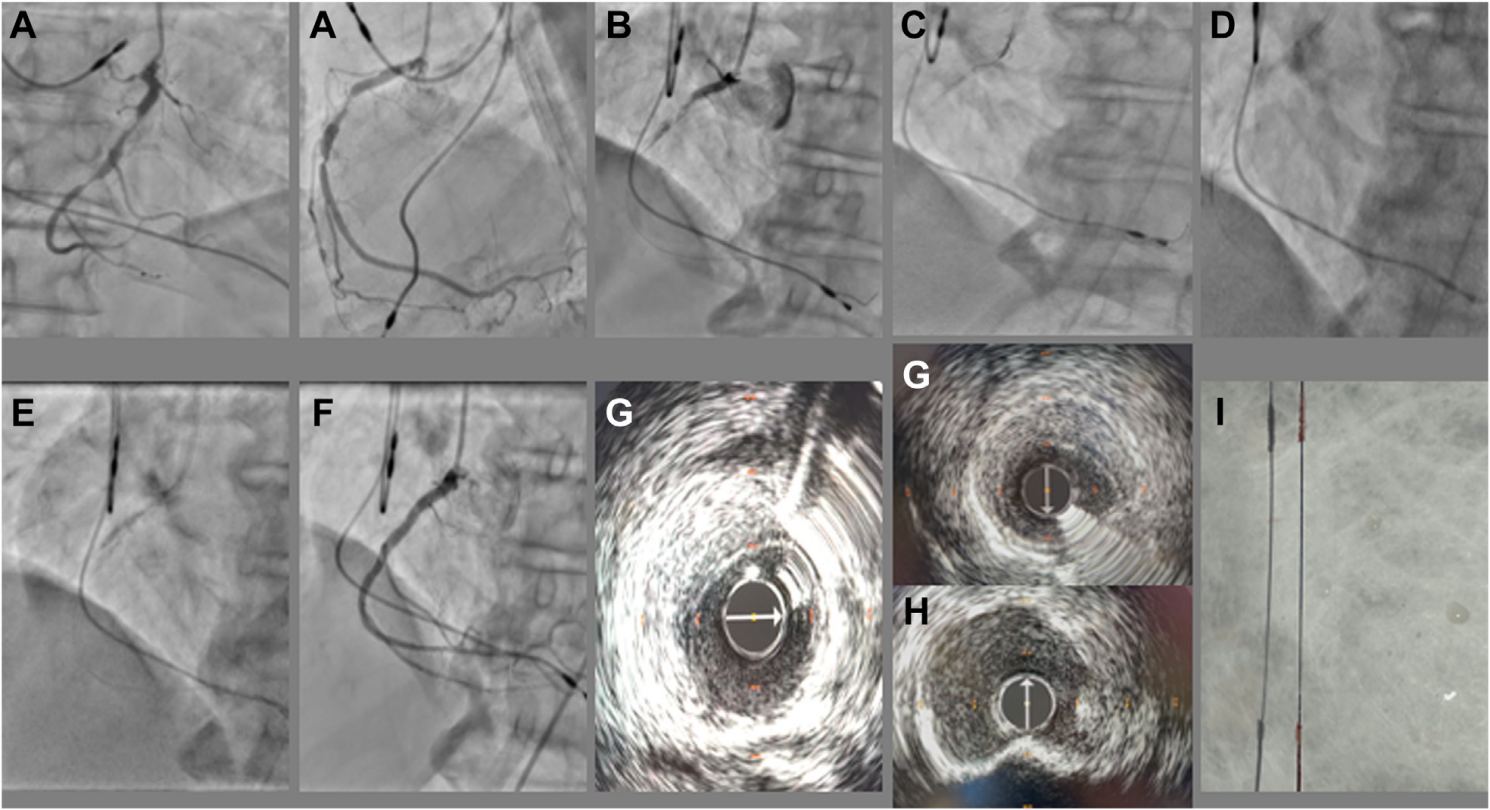
Coronary Angiograms **(A)** Initial diagnostic angiogram. **(B)** Ruptured balloon in the proximal right coronary artery (RCA). **(C)** Unable to advance guidewire. Balloon fragment obstructing the proximal RCA. **(D)** Subintimal wiring and subintimal tracking and reentry. **(E)** Subintimal plaque modification, with 2.5-mm noncompliant balloon inflated in the subintimal space, attempting to dislodge entrapped balloon. **(F)** Final angiographic result. **(G)** Intravascular ultrasound (IVUS) image demonstrating the crescent-shaped subintimal hematoma. **(H)** Post angioplasty IVUS demonstrating a calcium nodule in the proximal RCA at the site of balloon entrapment. **(I)** Ruptured balloon fragments glued to the wire.

An 82-year-old man was referred for coronary angiography (CAG) in the context of non-ST-segment elevation myocardial infarction. A nuclear medicine scan demonstrated moderate ischemia of the inferior wall. A CAG performed via a right radial artery demonstrated a severely calcified stenosis on the proximal and mid right coronary artery (RCA), with a calcific nodule (Figure 1A, Videos 1A and 1B). The left coronary artery had nonsevere coronary artery disease. Decision was taken to undergo percutaneous coronary intervention (PCI) of the proximal and mid RCA. A Balance middleweight II wire (Abbott) was advanced easily into the posterior descending artery. Initially, balloon angioplasty of the proximal RCA was performed with a semi-compliant 2.5 × 20-mm balloon inflated at 14 atmospheres, which was the rated burst pressure, but the balloon ruptured (Figure 1B, Video 2). Subsequent angiography demonstrated that the balloon was partially deflated and there was Thrombolysis In Myocardial Infarction (TIMI) flow 2 antegrade. Despite this, the patient remained hemodynamically stable.

**Learning Objectives**

• To perform a meticulous management algorithm for balloon entrapment during percutaneous coronary intervention to improve patient outcomes.
• To describe the subintimal calcium modification technique as a bailout strategy in the case of balloon entrapment.
• To promote the importance of lesion preparation with adequate calcium modification techniques to avoid these complications.

## Past Medical History

The patient had no significant cardiac or extracardiac medical history.

## Investigations

We attempted to remove the balloon by forceful traction, but the distal marker started lengthening as we pulled further. We were able to partially pull the balloon into the guiding catheter, but neither the balloon nor the wire could be removed further. A balloon fragment was still present in the mid RCA on angiography. The patient developed chest pain at this point and the antegrade flow was TIMI flow grade 1.

## Management

We cut the balloon shaft and advanced a 5.5-F Guideliner (Teleflex), with deep intubation of our guiding catheter, but the guiding extension was unable to advance past the balloon fragment in the mid RCA. Further, directed pulling through the guide extension catheter did not allow the retrieval of the entrapped balloon. Subsequently, we obtained a right femoral artery access to advance another 6-F Judkins Right guiding catheter to perform the Ping-Pong technique^1^ (“use of 2 guide catheters into the same coronary artery”). We were unable to advance a guidewire into the true lumen, as the balloon fragment was obstructing the stenosed segment mid RCA (Figure 1C, Video 3). At this point, the usual described maneuvers for retraction of entrapped gear had been attempted with no success. We decided that advanced chronic total occlusion (CTO) techniques were necessary. The initial plan was to obtain subintimal access proximal to the entrapped balloon and try a distal reentry and external plaque crush to dislodge the balloon.

A guidewire was used to access the subintimal space proximal to the entrapped gear, followed by a microcatheter Turnpike LP (Teleflex) and was advanced beyond the entrapped balloon. An attempt to reenter the true lumen was performed at this stage with a Gaia 3 Next wire (Asahi Intecc), but failed. A Gladius Mongo 14 (Asahi Intecc) was advanced into the subintimal space at the mid vessel, and a subintimal tracking and reentry was attempted but was also unsuccessful (Figure 1D, Video 4).

We thought of using a String-ray balloon (Boston Scientific), yet we wanted to avoid the use contrast, which would have caused large proximal dissection planes. Subintimal plaque modification and external plaque crush were the last remaining options to dislodge the entrapped balloon. We predilated the subintimal space with a 2.0- and 2.5-mm noncompliant balloon to 16 atmospheres (Figure 1E, Video 5). Afterward, further pulling on the gear was successful in retrieving all entrapped balloon fragments as well as the coronary guidewire. Rewiring of the native vessel with Sion Blue frontline guidewire (Asahi Intecc) was performed. Wire was successfully delivered to the right ventricular marginal branch. A dual-lumen catheter was advanced to the branch and a second frontline guidewire was delivered carefully in the distal segment of the RCA. Predilatation and stent implantation was performed successfully with 4 drug-eluting stents.

Intravascular ultrasound was performed using the Refinity system (Philips) and demonstrated a subintimal hematoma distally, a well-expanded and well-apposed stent, and circumferential calcium in the mid and proximal RCA, with a calcium nodule at the site of the balloon rupture and entrapment. The final CAG showed TIMI flow grade 3 antegrade in the RCA (Figure 1F, Video 6).

## Discussion

In this report, we describe the use of a subintimal plaque modification technique by side-ballooning the subintimal space, which is the first time this bailout strategy was undertaken to manage this rare complication. The incidence of entrapped material during PCI is reported at a rate between 0.4% and 1.0%, and the incidence of balloon entrapment is even lower.

However, despite this rare occurrence, entrapment of a balloon is a potentially fatal complication and must be managed urgently. We reviewed the literature by searching PubMed for occurrences of balloon entrapment using the keywords “balloon entrapment,” “balloon rupture,” and “undeflatable balloon” and found 21 cases highlighting this complication (Supplemental Table 1). Use of subintimal calcium modification technique for retrieval of an entrapped balloon has never been described. Most of these cases occurred in the left anterior descending artery (n = 9 of 21; 42.8%) and in the RCA (n = 8 of 21; 38.1%) (Supplemental Table 1). Factors leading to entrapment reported in the literature include tortuous, angulated, and calcified lesions, with sharp calcium nodules.^2^ In addition, inflating beyond the rated burst pressure has been suggested as a risk factor for this complication.^3^ In our case, preangioplasty intracoronary imaging could have better showcased the calcified nodule, which was not appreciated on coronary angiography. Furthermore, we inflated the balloon at rated burst pressure. Performing balloon angioplasty using a noncompliant balloon or rotational atherectomy as our initial strategy in this calcified lesion could have avoided this complication. Another preventive strategy would have been to size the initial dilating balloon to the reference diameter, as an undersized balloon increases the risk for this complication. The most popular techniques for retrieval of entrapped balloons described in the literature involved deep intubation of a large guiding catheter in 4 of 21 (19.0%) (Supplemental Table 1) cases and emergent cardiac surgery in 8 cases (n = 8 of 21; 38.1%) (Supplemental Table 1). Rarely, snaring, balloon stent crush, manual retrieval by pulling strongly, and buddy balloon technique have also been used as potential bailout strategies to this rare but potentially fatal complication. Previously, we described the technique of external subintimal plaque modification for management of a stuck rotational atherectomy burr.^4^ This technique has initially been described in CTO PCI to manage balloon uncrossable and undilatable lesions.^5^ These CTO skills should be kept in mind as a valuable bailout strategy when dealing with entrapped material in the coronary arteries, thus avoiding potentially morbid urgent surgery.

## Follow-Up

The patient was discharged 3 days later, after an uneventful postprocedural course. Peak troponin T Hs level was reached the next day (659 ng/L).

## Conclusions

The subintimal plaque modification technique is a bailout strategy for balloon entrapment and should be kept in mind in these critical situations.

## Funding Support and Author Disclosures

Dr Avram is supported by the Fonds de Recherche du Québec en Santé (FRQS, Grant Number 312758); and has received speaker fees from Servier Canada, Boehringer Ingelheim and the Quebec Cardiologist Association. All other authors have reported that they have no relationships relevant to the contents of this paper to disclose.

## Appendix

Supplemental Video 1 Initial diagnostic angiogram AP Cranial 20°.

Supplemental Video 2 Ruptured balloon in the proximal RCA.

Supplemental Video 3 Unable to advance guidewire in the true lumen.

Supplemental Video 4 Subintimal wiring and subintimal tracking and reentry (STAR).

Supplemental Video 5 Subintimal plaque modification, with 2.5 mm NC balloon inflated in the subintimal space, attempting to dislodge entrapped balloon.

Supplemental Video 6 Final angiographic result.

## References

[1] Brilakis E.S., Grantham J.A., Banerjee S. (2011). “Ping-pong” guide catheter technique for retrograde intervention of a chronic total occlusion through an ipsilateral collateral. Catheter Cardiovasc Interv.

[2] Gasparini G.L., Sanchez J.S., Regazzoli D. (2021). Device entrapment during percutaneous coronary intervention of chronic total occlusions: incidence and management strategies. EuroIntervention.

[3] Chang W.T., Chen J.Y., Li Y.H., Tsai L.M., Lee C.H. (2013). A two-case series of entrapment of a ruptured balloon in the coronary artery: avoidable complications and nonsurgical management. J Formos Med Assoc.

[4] Sayah N., Rey F., Nosair M., Ly H. (2019). Stuck between a rock and a hard place: management of an entrapped rotablator burr. J Am Coll Cardiol Case Rep.

[5] Hall A.B., Brilakis E.S. (2019). Hybrid 2.0: subintimal plaque modification for facilitation of future success in chronic total occlusion percutaneous coronary intervention. Catheter Cardiovasc Interv.

