# Supplementary material for: Get Out of Jail: Managing an Entrapped Balloon Using Subintimal Plaque Modification
Source: JACC Case Rep. 2022 Oct 5;4(19):1252–5. doi: 10.1016/j.jaccas.2022.07.004 (PMC9666741; doi:10.1016/j.jaccas.2022.07.004)
Supplement: Supplemental Data [file mmc7.docx]

**Supplemental Table 1. Systematic review of entrapped balloons over the past decades**

| **Patient no** | **Age** | **Complication** | **Artery** | **Management technique** |
| --- | --- | --- | --- | --- |
| 1^1^ | 78 | Rupture | RCA | Deep intubation SCR 3.5 7F Guiding catheter |
| 2^1^ | 48 | Rupture | LAD | Deep intubation EBU 7F |
| 3^2^ | 45 | Non-deflated | LAD | Deep intubation 6F JR4 and the use of a stiff end of a 0.018 wire |
| 4^3^ | 72 | Entrapped cutting balloon | Anomalous RCA | Deep engagement 6F JR4 |
| 5^4^ | 69 | Rupture cutting balloon | Iliac artery | Snare |
| 6^5^ | 65 | Rupture | RCA | Goose neck Microsnare retrieval system |
| 7^6^ | 71 | Non-deflated | RCA | Surgery |
| 8^7^ | 69 | Rupture | LCx | Surgery |
| 9^8^ | 78 | Entrapped | LAD | Surgery |
| 10^8^ | 31 | Entrapped | LAD | Surgery |
| 11^8^ | 50 | Entrapped | LAD | Surgery |
| 12^9^ | 65 | Entrapped | RCA | Failed surgical removal |
| 13^10^ | 69 | Rupture | LAD | Trapping balloon technique |
| 14^11^ | 65 | Entrapped under stent strut | LCx | Multiple wire entanglement |
| 15^12^ | 67 | Entrapped | RCA | Surgery |
| 16^13^ | 81 | Rupture | LAD | Surgery |
| 17^14^ | 77 | Rupture | RCA | Not recovered/ Rotational atherectomy and stenting |
| 18^15^ | 74 | Entrapped | RCA | Manual Retrieval |
| 19^16^ | 68 | Non-deflated | LM | Balloon stent crush |
| 20^17^ | 73 | Entrapped fragment | LAD | Rotational atherectomy |
| 21^18^ | 75 | Entrapped | LAD | Buddy balloon technique |

References

1. Chang WT, Chen JY, Li YH, Tsai LM, Lee CH. A two-case series of entrapment of a ruptured balloon in the coronary artery: Avoidable complications and nonsurgical management. J Formos Med Assoc [Internet]. 2015 Nov 1 [cited 2022 Apr 23];114(11):1135–9. Available from: https://pubmed.ncbi.nlm.nih.gov/23791003/

2. Girish MP, Gupta MD, Tyagi S. Entrapped coronary angioplasty stent balloon due to nondeflation: percutaneous retrieval by a simple technique. Catheter Cardiovasc Interv [Internet]. 2011 Jan 1 [cited 2022 Apr 23];77(1):58–61. Available from: https://pubmed.ncbi.nlm.nih.gov/20506291/

3. Blackman D, Dzavik V. Inadvertent detachment of an entrapped Cutting Balloon from the balloon catheter during treatment of in-stent restenosis. J Invasive Cardiol [Internet]. 2005 Nov [cited 2022 Apr 23];17(11). Available from: https://pubmed.ncbi.nlm.nih.gov/16264212/

4. Braun MA, Smith SJ, Merrill TN. Contralateral loop snare removal of a ruptured and entrapped angioplasty balloon. Cardiovasc Intervent Radiol [Internet]. 1996 [cited 2022 Apr 23];19(6):428–30. Available from: https://pubmed.ncbi.nlm.nih.gov/8994710/

5. Removal of a ruptured, detached, and entrapped angioplasty balloon after coronary stenting - PubMed [Internet]. [cited 2022 Apr 23]. Available from: https://pubmed.ncbi.nlm.nih.gov/10731273/

6. Breisblatt WM. Inflated balloon entrapped in a calcified coronary stenosis. Cathet Cardiovasc Diagn [Internet]. 1993 [cited 2022 Apr 23];29(3):224–8. Available from: https://pubmed.ncbi.nlm.nih.gov/8402847/

7. Nishiwaki N, Kawano Y, Furukawa K, Nakayama Y. [A case report of entrapment of PTCA balloon catheter caused by its rupture]. Nihon Kyobu Geka Gakkai Zasshi [Internet]. 1991 [cited 2022 Apr 23];39(8):1226–30. Available from: https://pubmed.ncbi.nlm.nih.gov/1940531/

8. Surgical management of entrapped percutaneous transluminal coronary angioplasty hardware - PubMed [Internet]. [cited 2022 Apr 23]. Available from: https://pubmed.ncbi.nlm.nih.gov/12484620/

9. Carell ES, Schroth G, Ali A. Circumferential balloon rupture and catheter fracture due to entrapment in a calcified coronary stenosis. Cathet Cardiovasc Diagn [Internet]. 1994 [cited 2022 Apr 23];32(4):346–8. Available from: https://pubmed.ncbi.nlm.nih.gov/7987916/

10. León Jiménez J, Roa Garrido J, Camacho Freire SJ, Díaz Fernández JF. Trapping as retrieval technique to resolve a ruptured and entrapped coronary balloon catheter. Catheter Cardiovasc Interv [Internet]. 2017 Nov 1 [cited 2022 Apr 23];90(5):773–6. Available from: https://pubmed.ncbi.nlm.nih.gov/28766865/

11. Kane JA, Gilchrist IC, Al-Sadawi M, Abdalamir M, Pyo R. A stepwise approach utilizing a double helix wire technique, snare capture and a switch from a transfemoral to transradial approach to remove an entrapped balloon. Cardiovasc Revasc Med [Internet]. 2021 Jul [cited 2022 Apr 23]; Available from: https://pubmed.ncbi.nlm.nih.gov/34238681/

12. Desai CK, Petrasko M, Steffen K, Stys T, Stys A. Retained Coronary Balloon Requiring Emergent Open Surgical Retrieval: An Uncommon Complication Requiring Individualized Management Strategies. Methodist Debakey Cardiovasc J [Internet]. 2019 Jan 1 [cited 2022 Apr 23];15(1):81–5. Available from: https://pubmed.ncbi.nlm.nih.gov/31049154/

13. Cuttone F, Saplacan V, Sabatier R, Buklas D. Angioplasty balloon catheter entrapment. Asian Cardiovasc Thorac Ann [Internet]. 2012 Aug [cited 2022 Apr 23];20(4):491. Available from: https://pubmed.ncbi.nlm.nih.gov/22879568/

14. Balasubramaniam K, Elbarouni B, Kass M, Minhas K, Ravandi A. Rotational Atherectomy in the Management of Ruptured and Entrapped Coronary Angioplasty Balloon. Cardiovasc Revasc Med [Internet]. 2021 Jul 1 [cited 2022 Apr 23];28S:140–3. Available from: https://pubmed.ncbi.nlm.nih.gov/33046415/

15. Leibundgut G, Degen C, Riede F. Transcutaneous Puncture of an Undeflatable Coronary Angioplasty Balloon Catheter. Case reports Cardiol [Internet]. 2018 Sep 3 [cited 2022 Apr 23];2018:1–5. Available from: https://pubmed.ncbi.nlm.nih.gov/30250754/

16. Omer AR, Bianco M, Lodhi A, Sattur S, Kaluski E. Angioplasty Balloon Entrapped Fully Inflated and Detached Within the Left Main Coronary Artery. Cardiovasc Revasc Med [Internet]. 2020 Nov 1 [cited 2022 Apr 23];21(11S):21–4. Available from: https://pubmed.ncbi.nlm.nih.gov/32088100/

17. Sawano S, Sakakura K, Tsurumaki Y, Fujita H. Entrapment of a completely radiolucent fragment of balloon catheter: should we try to retrieve or knock the invisible fragment? Cardiovasc Interv Ther [Internet]. 2021 Jul 1 [cited 2022 Apr 23];36(3):386–8. Available from: https://pubmed.ncbi.nlm.nih.gov/32557340/

18. Minami Y, Meguro K, Shimohama T, Yanagisawa T, Kakizaki R, Tojo T, et al. Successful Retrieval of Entrapped Balloon With Optical Coherence Tomography Guidance. JACC Cardiovasc Interv [Internet]. 2016 Oct 10 [cited 2022 Apr 23];9(19):2070–1. Available from: https://pubmed.ncbi.nlm.nih.gov/27639902/
